# Active Return-to-Center Control Based on Torque and Angle Sensors for Electric Power Steering Systems

**DOI:** 10.3390/s18030855

**Published:** 2018-03-14

**Authors:** Pan-Pan Du, Hao Su, Gong-You Tang

**Affiliations:** College of Information Science and Engineering, Ocean University of China, Qingdao 266100, China; dp1987829@163.com (P.-P.D.); gtang@ouc.edu.cn (G.-Y.T.)

**Keywords:** electric power steering systems, torque sensor, angle sensor, state switch, active return-to-center control

## Abstract

This paper presents a complete control strategy of the active return-to-center (RTC) control for electric power steering (EPS) systems. We first establish the mathematical model of the EPS system and analyze the source and influence of the self-aligning torque (SAT). Second, based on the feedback signals of steering column torque and steering wheel angle, we give the trigger conditions of a state switch between the steering assist state and the RTC state. In order to avoid the sudden change of the output torque for the driving motor when the state switches frequently between the steering assist state and the RTC state, we design an undisturbed state switching logic algorithm. This state switching logic algorithm ensures that the output value of the RTC controller is set to an initial value and increases in given steps up to a maximum value after entering the RTC state, and the output value of the RTC controller will reduce in given steps down to zero when exiting the RTC state. This therefore ensures smooth switch control between the two states and improves the driver’s steering feeling. Third, we design the RTC controller, which depends upon the feedback signals of the steering wheel angle and the angular velocity. In addition, the controller increases the auxiliary control function of the RTC torque based on vehicle speed. The experimental results show that the active RTC control method does not affect the basic assist characteristics, which effectively reduces the residual angle of the steering wheel at low vehicle speed and improves the RTC performance of the vehicle.

## 1. Introduction

Electric power steering (EPS) systems have the advantages of safety, energy saving, and environmental protection. It has become the mainstream of power steering technology for passenger cars because of its important role in the enhancement of handling stability and safety [1]. The EPS system adds a motor and deceleration mechanism on the basis of the traditional mechanical steering mechanism. Therefore, the friction of the steering system will increase and the return-to-center (RTC) performance will reduce.

In the running process of a vehicle, when the driver manually turns the steering wheel back to center, it often needs to correct the direction substantially or frequently. The vehicle’s driver cannot get a comfortable control feeling and is prone to fatigue, thus impacting traffic safety.

When the driver turns the steering wheel to center, the assist motor can apply the corresponding RTC torque to the steering system, which can solve the problem that the steering wheel cannot return to center because of the friction of the mechanical parts, and makes the operation track of the steering wheel and the running track of the vehicle the same, thus greatly reducing the burden on, and providing a comfortable and stable driving experience for, the driver [2].

Recently, experts and scholars at home and abroad have done a lot of research on the RTC control of EPS systems. For EPS systems with a steering wheel angle sensor installed, since the absolute position of the steering wheel can be obtained in real-time, the research on this kind of system mainly focuses on the realization method of RTC control. This research takes the steering wheel angle as an input signal, and mainly adopts the Proportional Integral Derivative (PID) control method [3] or other improved PID algorithm [4,5] to make the steering wheel return to center.

Because the price of an angle sensor is higher, in order to save costs many researchers use motor voltage and current to estimate motor speed, and then estimate the steering wheel angle through integration and after that, torque estimation [6], test data statistics [7], RTC torque compensation [8,9] and other methods are used to realize RTC control—these methods have a certain effect on the RTC ability of the EPS systems. However, it depends on the precision of the system model and brings great uncertainty to the RTC control due to the lack of steering wheel angle information. Moreover, the algorithm is complex and is prone to problems and difficult to promote.

For vehicles equipped with an electronic stability program (ESP), the relevant vehicle traveling state information can be obtained from the sensor signals of the ESP such as vehicle sideslip angle and yaw rate, and the RTC torque can be estimated based on these signals in order to realize RTC control [10,11], but these methods do not apply to vehicles without ESP installed. 

With the development of sensor technology, a composite sensor is used to detect the torque and angle of the steering wheel, and the price of these is gradually decreasing. The steering system is vital to the safety of the car; therefore, it is not worth sacrificing safety to save costs and the active RTC control with angle sensor has gradually become mainstream [12,13,14].

At present, the research on the active RTC control of EPS systems is mainly focused on the control method. There is less research on the determination method of the RTC state. The most common method for determining the RTC state is to judge whether the steering wheel angle and the angular velocity are in the same direction—good results can be achieved for the “release-return” state. For the complex steering condition in which power steering state and RTC state switch frequently, however, RTC torque will have a certain impact on the driver’s feel because the angular velocity of the steering wheel cannot be mutated [15,16,17].

When the assist state and the RTC state are switching because the direction of the assist torque and the RTC torque are opposite one another, if they are not connected properly, the steering process will result in a sense of frustration and affect the feel for the driver. It is therefore important for RTC control, but to the best of the authors’ knowledge, no literature has been published in this regard.

The self-aligning torque of the vehicle increases with the increase of vehicle speed [18], therefore, the RTC torque should vary with the vehicle speed. Most research focuses on the RTC performance at a fixed vehicle speed but does not pay attention to the influence of vehicle speed. 

This paper considers the deficiency of current RTC control for EPS systems, and a method based on steering wheel angle signal is proposed to accurately determine the RTC state of the vehicle. An RTC control strategy for the EPS system is designed to ensure uniformly smooth transition of the torque for the steering wheel to avoid affecting the feel for the driver.

## 2. Model of EPS Systems

**Figure 1:**
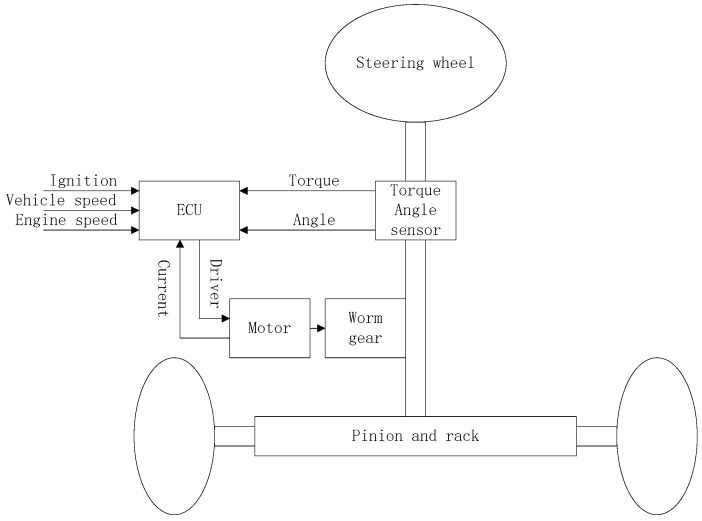
Mechanical structure of Electric Power Steering (EPS) systems.

The mechanical structure of EPS systems is shown in Figure 1, which mainly composes of torque-angle sensor, steering wheel, steering column, worm gear, rack and pinion, the assist motor, the electronic control unit (ECU), and so forth.

Its basic operating principle can be summarized as follows: when the vehicle is in the starting or running state, the input torque and the angle of the steering wheel are detected through the torque-angle sensor placed on the steering column, and these signals will be transmitted to an ECU for processing, and then drive the assist motor to provide an assist torque or a RTC torque [19,20].

The dynamic model of an EPS system is governed by the relationship between steering mechanism, the electric motor dynamic behavior and the road/tire contact forces. From Figure 1, following Newton’s second law, a nonlinear dynamic model of the EPS system can be derived. The mathematical model of the EPS system can be described as:

(1) $$J_{s}{\ddot{\theta }}_{s}=T_{d}-T_{s}-B_{s}{\dot{\theta }}_{s}$$

(2) $$T_{s}=K_{s}(\theta_{s}-\theta_{p})$$

(3) $$J_{m}{\ddot{\theta }}_{m}=T_{m}-K_{m}(\theta_{m}-G\frac{p}{r_{p}})-B_{m}{\dot{\theta }}_{m}$$

(4) $$M_{r}\ddot{p}+B_{r}\dot{p}+T_{SAT}+F_{fric}\mathrm{sgn}(\dot{p})=(T_{s}+T_{s}G)/r_{p}$$

where $J_{s}$ is the steering column moment of inertia; $\theta_{s}$ is the rotation angle of the steering wheel; $T_{d}$ is the steering wheel torque; $T_{s}$ is the measurement of the torque sensor; $B_{s}$ is the steering column viscous damping; $K_{s}$ is the steering column stiffness; $\theta_{p}$ is the rotation angle of the pinion; $J_{m}$ is the motor moment of inertia; $\theta_{m}$ is the rotation angle of the motor shaft; $T_{m}$ is the electromagnetic torque provided by electric motor; $K_{m}$ is the motor stiffness; $B_{m}$ is the motor viscous damping; G is the motor gear ratio; p is the rack position; $r_{p}$ is the pinion radius; $M_{r}$ is the mass of the rack; $B_{r}$ is the rack viscous damping; $T_{SAT}$ is the self-aligning torque of the vehicle; $F_{fric}$ is the friction torque of the steering system.

**Figure 2:**
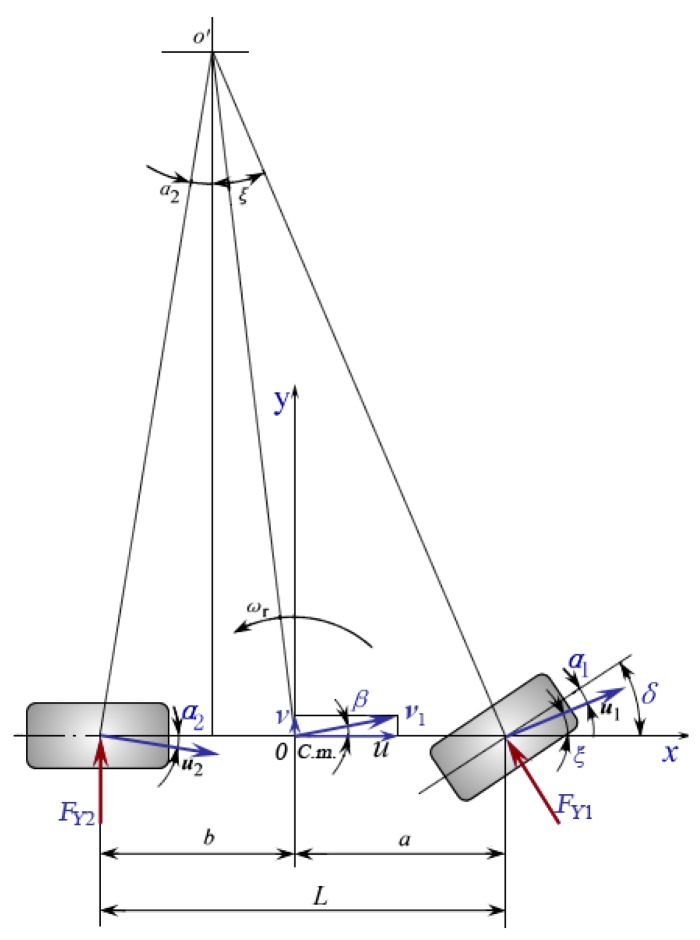
Bicycle model.

In order to analyze the lateral dynamics of the vehicle and estimate the self-aligning torque (SAT) of the front wheels, a bicycle model is used. The principle sketch map of the bicycle model is shown in Figure 2. This model includes several important exclusions and simplifications. These simplifications greatly reduce the model’s complexity and degrees of freedom, but do not significantly affect the vehicle lateral dynamics.

The lateral force is a nonlinear function of the wheel’s slip angle. However, when a car is in the normal running state, the slip angle is very small; therefore, the lateral force can be approximated with a linear function of slip angle. The front and rear lateral force of the wheels is:

(5) $$\begin{array}{l} F_{Y1}=K_{Y1}\alpha_{1}\\ F_{Y2}=K_{Y2}\alpha_{2} \end{array}$$

where $F_{Y1}$ and $F_{Y2}$ are front and rear lateral force of the wheels, $K_{Y1}$ and $K_{Y2}$ are front and rear tire cornering stiffness, $\alpha_{1}$ and $\alpha_{2}$ are front and rear slip angle of the wheels.

According to the coordinate relation of Figure 2,

(6) $$\begin{array}{l} \alpha_{1}=-(\delta -\xi )=\beta +\frac{a\omega_{r}}{u}-\delta \\ \alpha_{2}=\frac{v-b\omega_{r}}{u}=\beta -\frac{b\omega_{r}}{u} \end{array}$$

where δ is the front wheel turning angle, ξ is the angle between the X-axis and the velocity at the midpoint of the front shaft, β is the sideslip angle of the vehicle’s center of mass, a and b are the distance from front and rear tire to vehicle’s center of mass, $\omega_{r}$ is the yaw rate of the vehicle’s center of mass, u and v are the longitudinal and latitudinal velocity of the vehicle at center of mass.

SAT is mainly generated by the reactive force of the front lateral force; because the front lateral force is proportional to the front slip angle, the SAT can be approximated with a linear function of the front slip angle.

(7) $$T_{SAT}=-K_{SAT}\alpha_{1}=K_{SAT}(\delta -\beta -\frac{a\omega_{r}}{u})$$

where $K_{SAT}$ is the stiffness of the self-aligning torque. It can be seen that SAT increases with an increase in vehicle speed.

In this paper, a permanent magnet brushless DC motor is used as the assist motor. The dynamic model of the motor is:

(8) $$L_{a}\frac{di(t)}{dt}+R_{a}i(t)=U_{a}(t)-E(t)$$

where $L_{a}$ is the electric inductance of the motor, $R_{a}$ is the electric resistance of motor, i(t) is the motor armature current, $U_{a}(t)$ is the motor armature voltage, E(t) is the back electromotive force of the motor armature.

According to the motor theory, the following expression is given:

(9) $$\begin{array}{l} E(t)=K_{e}(\Phi )n(t)\\ T_{m}(t)=K_{t}(\Phi )i(t) \end{array}$$

where Φ is the magnetic field strength of the motor stator, $K_{e}(\Phi )$ and $K_{t}(\Phi )$ are positive number dependent on the magnetic field strength Φ, n(t) is speed of the motor shaft, $T_{m}(t)$ is the motor torque. Assuming that Φ is constant, then $K_{e}(\Phi )$ and $K_{t}(\Phi )$ are constants, and denoted as $K_{e}$ and $K_{t}$, respectively [21,22].

The motor torque $T_{m}(t)$ is applied to the steering column through the worm gear reducer, $T_{a}(t)$ is the torque acting on the steering column and can be expressed as:

(10) $$T_{a}(t)=K_{r}T_{m}(t)$$

where $K_{r}$ is reduction ratio of the worm gears.

The torque acting on the steering wheel is:

(11) $$T_{d}(t)=T_{r}(t)-T_{a}(t)$$

where $T_{d}(t)$ is the torque acting on the steering wheel and $T_{r}(t)$ is the resistance torque of the steering column caused by friction between the tire and the ground.

## 3. Active RTC Control Strategy

There are two important aspects needed to solve the active RTC control problem of EPS systems; one is the RTC state judgment, and the second is the RTC algorithm. The primary goal of EPS systems is to realize power steering and the active RTC is used to improve the performance of the vehicle and should not affect the basic assist characteristics of EPS systems.

### 3.1. RTC State Determination

Active RTC control does not work in the steering assist process and it is triggered only when the vehicle enters the RTC state. Therefore, it is a precondition of the active RTC control to accurately judge the current running state of the steering system.

The most commonly used method is based on the sign of steering wheel angle and angular velocity—when $\theta \cdot \dot{\theta }<0$, it goes back to the RTC state. However, this method does not accurately determine the operation intention of the driver because in practice, a steering process is not a simple “release-return” process. The steering wheel may be returning, but because of the driver’s intervention, the steering wheel will remain stationary or run with regular power steering, and the assist state and the RTC state will be switched frequently. Because the angular velocity of the steering wheel cannot be changed suddenly, the disturbance switch will be generated. Disturbance torque caused by disturbance switching can affect the driver’s feel. Therefore, it is necessary to use steering torque signal to detect whether or not the driver has as an intention to change from the RTC state to a steering state. 

In the power steering control state, when the steering torque exceeds a setting torque value $T_{d0}$, the assisted motor will provide assist torque, that is to say, if the steering torque exceeds the setting torque value $T_{d0}$, it means that the driver has an intention to turn the steering wheel. If there is such an intention, it is determined that the RTC control state is switched to the power steering control state.

When the vehicle is running at low speed or is at rest, it is not necessary and not suitable to use active RTC control. When driving at high speed, due to the small steering resistance, the passive RTC torque of the car is sufficient to make the steering wheel return to center, so it does not need active RTC control. Moreover, it may even need damping control at the time of return. Therefore, active RTC control is only executed within a set vehicle speed range. The vehicle does not need active RTC control when it stops, so the minimum vehicle speed $V_{\min}$ is usually set to 0 Km/h. According to Equation (7), as vehicle speed increases SAT increases, when the vehicle speed exceeds a certain value, the SAT can make the steering wheel return to center so it does not need active RTC control at this time, and this certain value is defined as the maximum vehicle speed $V_{\max}$, $V_{\max}$ can be determined by a real vehicle test.

Based on the above analysis, the judgment algorithm of the RTC state put forward is as follows.

1. Vehicle speed should be in a certain range, that is, $V_{\min}<V<V_{\max}$.
2. The torque $T_{d}$ acting on the steering wheel is less than a certain threshold $T_{d0}$, that is, $\left|T_{d}\right|<T_{d0}$.
3. The sign of steering wheel angle θ and angular velocity $\dot{\theta }$ is opposite, that is, $\theta \cdot \dot{\theta }<0$.
4. The steering wheel angle θ is greater than the preset dead zone Δ, that is, |θ|>Δ.

**Figure 3:**
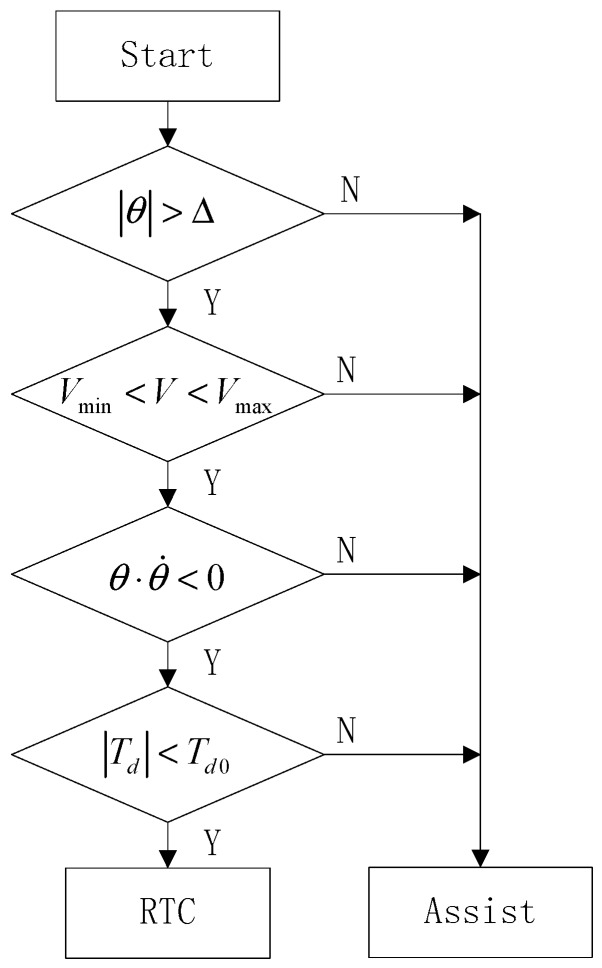
Flow chart of judging state switch.

The flow chart of judging state switch is shown in Figure 3.

### 3.2. Control System Structure

**Figure 4:**
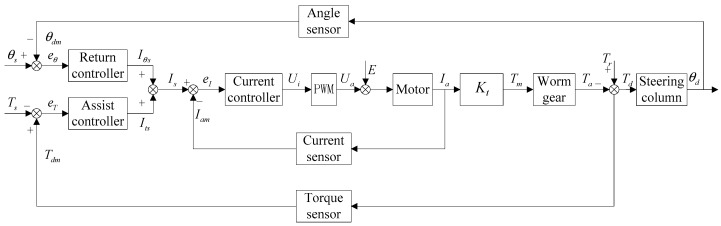
Structure of the EPS control system.

The structure of the EPS control system proposed in this paper is shown in Figure 4.

When the driver turns the steering wheel, the EPS controller gets steering wheel torque $T_{d}(t)$ and steering wheel angle $\theta_{d}(t)$ through a torque-angle sensor placed on the steering column. The measured values of the torque and angle are used as inputs to the assist controller and RTC controller.

The EPS controller uses the desired steering wheel torque $T_{s}(t)$ and the actual measurement of the torque signal $T_{dm}(t)$ to calculate the error of the torque control loop:

(12) $$e_{T}(t)=T_{dm}(t)-T_{s}(t)$$

The EPS controller uses the desired steering wheel angle $\theta_{s}(t)$ and the actual measurement of the angle signal $\theta_{dm}(t)$ to calculate the error of the angle control loop:

(13) $$e_{\theta }(t)=\theta_{dm}(t)-\theta_{s}(t)$$

The sum of the output of the assist controller and the output of the RTC controller is used as the input of the current controller:

(14) $$I_{s}(t)=I_{ts}(t)+I_{\theta s}(t)$$

The EPS controller uses the output current of the motor $I_{a}(t)$ and the measurement of the motor current $I_{am}(t)$ to calculate the error of the current control loop:

(15) $$e_{I}(t)=I_{s}(t)-I_{am}(t)$$

The torque acting on the steering wheel can be obtained by:

(16) $$T_{d}(t)=T_{r}(t)-T_{a}(t)$$

where $T_{d}(t)$ is the torque acting on the steering wheel and $T_{r}(t)$ is the resistance torque of the steering system.

### 3.3. RTC Control Strategy

When the torque $T_{d}$, acting on the steering wheel, is less than a certain threshold $T_{d0}$, that is $\left|T_{d}\right|<T_{d0}$, the steering system will put RTC force on the steering wheel. When the value of $T_{d0}$ is smaller (generally 2–3 (N.m) for a car), small changes in torque may affect the driver’s feel, especially when the assist state and the RTC state are switching. If the RTC torque and the assist torque are not connected properly, the steering process will result in a sense of frustration and affect the driver’s feel.

**Figure 5:**
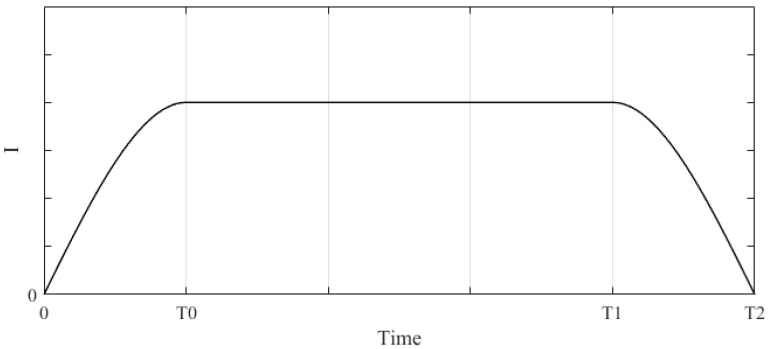
**Return-to-Center** (RTC) process.

In order to avoid sudden changes in the torque affecting the driver’s feel, we divide a complete RTC process into three stages. The RTC process is shown in Figure 5, where 0~T0 is the gradually increasing stage of RTC torque, T0~T1 is the maintenance stage of RTC torque, T1~T2 is the gradually reducing stage of RTC torque.

At the beginning of the RTC state, the RTC torque will increase gradually, the RTC torque will maintain its value when it increases to the set value, and when exiting the RTC state (close to center or entering the assist state), the RTC torque will reduce gradually, thus ensuring that the torque is uniformly smooth.

When switching from the assist state to the RTC state, the output value of the RTC controller is $I_{\theta s1}$ when the measured RTC torque is the smallest (less than the torque that the driver can perceive, usually 0.3 (N.m)), and this value is set to the output’s lower limit; the output value of the RTC controller is $I_{\theta s2}$ when the measured RTC torque is the largest, and this value is set to the output’s upper limit. The output step of the RTC controller is $\Delta I_{\theta s}$. $I_{\theta s1}$, $I_{\theta s2}$ and $\Delta I_{\theta s}$ need road testing to be measured.

After entering the RTC state, the output value of the RTC controller is set to $I_{\theta s1}$, and then increased in step $\Delta I_{\theta s}$ until reaching the maximum value.

(17) $$I_{\theta s}(t)=\left\{ \begin{array}{ll} I_{\theta s1}+\sum \Delta I_{\theta s}, & I_{\theta s1}\leq I_{\theta s}(t)\leq I_{\theta s2}\\ I_{\theta s2}, & I_{\theta s}(t)>I_{\theta s2} \end{array}\right.$$

When exiting the RTC state, the output value of the RTC controller will reduce in step $\Delta I_{\theta s}$ until zero.

(18) $$I_{\theta s}(t)=\left\{ \begin{array}{ll} I_{\theta s2}-\sum \Delta I_{\theta s}, & 0\leq I_{\theta s}(t)\leq I_{\theta s2}\\ 0, & I_{\theta s}(t)<0 \end{array}\right.$$

**Figure 6:**
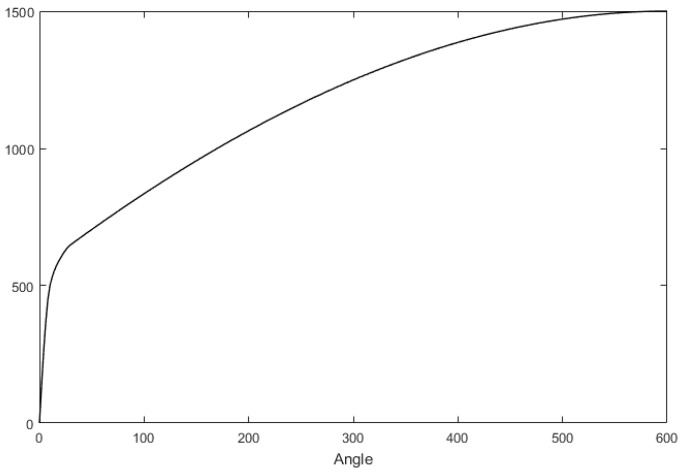
Relationship between the RTC torque and the steering wheel angle.

According to Equation (7), at the same vehicle speed, the SAT increases with the front wheel turning angle, that is to say, the return speed of the steering wheel increases with the angle of the steering wheel, which is consistent with the driver’s driving habits. In order to keep this driving habit, the output value of the RTC controller $I_{\theta s2}$ should increase with the steering wheel angle. In order to avoid the overshoot when the steering wheel is near the center, $I_{\theta s2}$ should reduce rapidly when approaching the center. The corresponding relationship between the RTC torque and the steering wheel angle is shown in Figure 6, and this curve can be determined by real vehicle tests.

**Figure 7:**
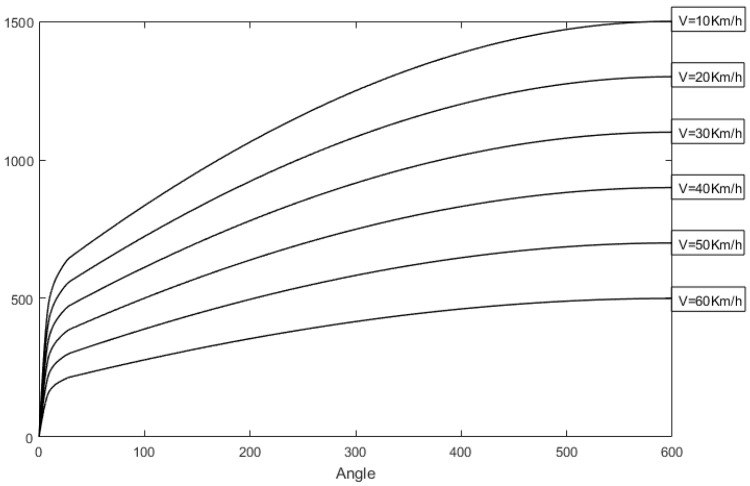
Corresponding relationship between the RTC torque and the vehicle speed.

The maximum output value of the RTC controller $I_{\theta s2}$ will also change based on the vehicle speed. The faster the speed, the smaller the RTC torque. The corresponding relationship curves between the RTC torque and the vehicle speed are shown in Figure 7.

## 4. Active RTC Control of Real Vehicle Testing

**Figure 8:**
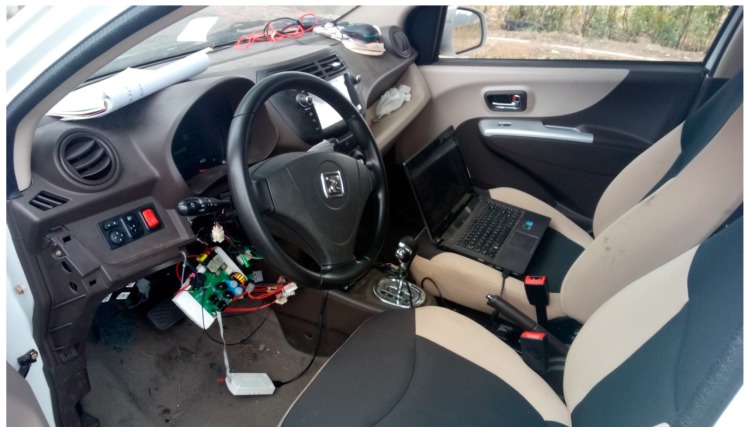
Experimental vehicle.

The test vehicle is an electric vehicle equipped with an EPS system as shown in Figure 8. In order to test the RTC control effect, the assist characteristic test is carried out first to investigate whether the active RTC control should affect the basic assist characteristics of EPS systems while improving the RTC performance of the vehicle, then test the RTC control effect and the effect of the RTC control algorithm on the RTC performance of the vehicle is investigated.

### 4.1. Assist Characteristic Testing

**Figure 9:**
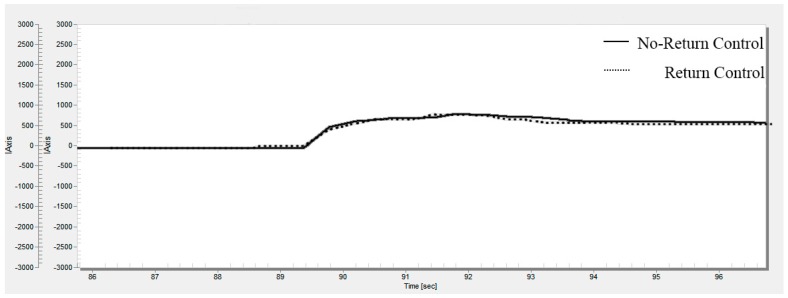
Assist characteristic test.

The steering torque curves of the steering wheel with active RTC control and passive RTC control are shown in Figure 9. From the comparison curves of the torque of the steering wheel, we see that the control effect of active RTC control is basically the same as that of passive RTC control. It is shown that the proposed method in this paper has no effect on the basic assist characteristics, and it will not affect the basic assist characteristics because of the addition of RTC control.

### 4.2. Active RTC Testing

**Figure 10:**
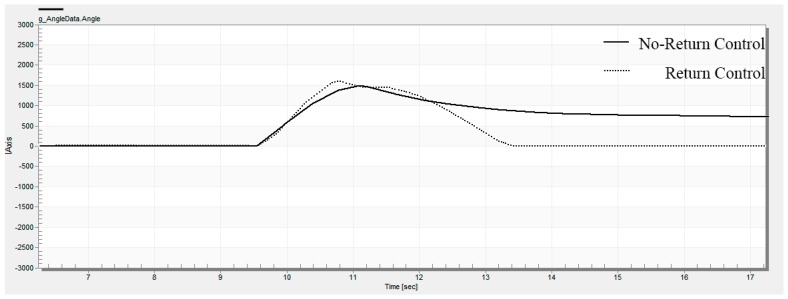
RTC curves of the steering wheel.

The driver drives the test car in a straight line and records the zero lines of the measured variables, then turns the steering wheel to 180°. After that, we fix the steering wheel angle, keep the vehicle speed steady, and start recording test data. After some time, the driver releases the steering wheel and records the relationship curve between steering wheel angle and time. The RTC curves of the steering wheel are shown in Figure 10.

**Figure 11:**
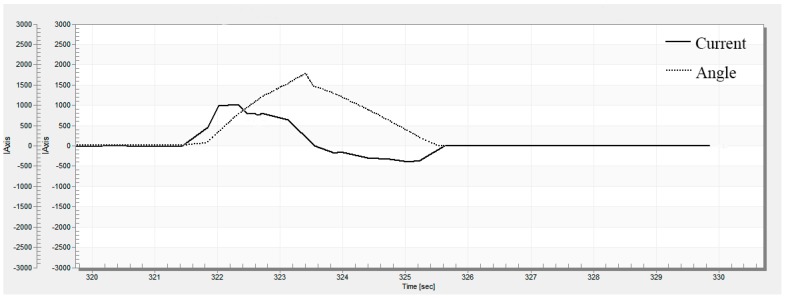
Motor’s current characteristic.

It can be seen from the test curve that the residual angle of the steering wheel can be reduced effectively after the active RTC control is added, and the RTC performance is improved. Figure 11 shows the motor’s current characteristic at the time of return. The driver turns the steering wheel to 180° and holds on, then releases the steering wheel. It can be seen from the curve of the motor current that the motor provides the assist current during the assist state. When entering the RTC state, the active RTC control method provides compensation current to help the steering wheel return to center. At the beginning of the RTC control, the RTC current increases gradually. As the steering wheel approaches the center, the RTC current decreases gradually. This is consistent with the control strategy we designed.

The RTC control test results show that the active RTC control method is only executed when the steering system is in a RTC state, and this method obviously reduces the residual angle of the steering wheel and effectively solves the problems of the lack of passive RTC torque at low vehicle speeds.

## 5. Conclusions

Through the analysis of the RTC process, the method of judging the RTC state of the vehicle is described in detail. Active RTC control is applied only when the steering system is in an RTC state to avoid affecting the driver’s feel. In order to avoid sudden changes in the torque, the RTC torque gradually increases and reduces, thus ensuring that the torque is uniformly smooth. The control strategy of the EPS system increases the RTC auxiliary control function based on the vehicle speed in addition to the feedback signals of steering wheel angle and angular velocity. The advantage of the proposed algorithm can be summarized in Table 1.

In order to verify the actual control effect of active RTC control, a real vehicle test is carried out. The experimental results show that the active RTC control method does not affect the basic assist characteristic, which effectively reduces the residual angle of the steering wheel at low vehicle speeds and improves the RTC performance of the vehicle.

## Figures and Tables

**Table 1:** Advantages of the algorithm.

Proposed Algorithm	Before	After
Add torque signal to judge the RTC state	1. It cannot accurately determine the operation intention of the driver. 2. The disturbance torque will be generated and affect the driver’s feel.	1. It can accurately determine the operation intention of the driver through the torque. 2. RTC control cannot affect the driver’s feel.
Undisturbed state switch	1. The RTC torque and the assist torque are not connected properly. 2. The steering process will result in a sense of frustration	1. It can avoid sudden changes in the torque. 2. The RTC torque gradually increases and reduces and makes the torque uniformly smooth.
RTC control with vehicle speed	RTC velocity of the steering wheel is too fast at high vehicle speed.	RTC velocity of the steering wheel will slow down at high vehicle speed.
